# Numerical Investigation of Overtopping Prevention for Optimal Safety Dike Design

**DOI:** 10.3390/ijerph192416429

**Published:** 2022-12-07

**Authors:** Namjeong Son, Yoojin Kim, Mimi Min, Seungho Jung, Chankyu Kang

**Affiliations:** 1 Department of Environmental Engineering, Ajou University, Suwon 16499, Republic of Korea; 2 Research Center for Safety and Health, School of Social Safety System Engineering, Hankyong National University, Anseong 17579, Republic of Korea

**Keywords:** dike, overtopping, VOF model, deflector plate

## Abstract

Leakage accidents at chemical facilities have a negative impact on both the environment and human life, and the government has established and implemented regulations on dikes in order to minimize such accidents. However, the overtopping phenomenon in which chemicals overflow the dike due to catastrophic leakage requires additional safeguards. In this study, the mitigation effect was confirmed by simulating tanks and dikes using various deflector plates to minimize the effect of spilled chemicals. ANSYS Fluent 19.1, a computational fluid dynamics program, was used, and the overtopping effect was compared with a dike design that satisfies the safety regulations using a volume of fluid (VOF) model that analyzes multiphase flow through a surface tracking technique. Nitric acid and sulfuric acid were used in the study; they were selected because they are frequently involved in leakage accidents. In the event of a leak in a liquid tank, a dike with a deflector plate was very effective in reducing overtopping, and a deflector at a 45° angle was more effective than a 30° deflector. However, it is necessary to install additional safeguards at the joint between the dike and the deflection plate to withstand the force of the liquid.

## 1. Introduction

The chemical processing industry (CPI) often deals with hazardous chemicals and/or processes that pose a risk of major accidents [1,2]. As the amount and production of chemical substances increases, the damage caused by hazardous chemical leakage accidents is increasing, and the concerns of nearby residents are also increasing [3,4]. Damage from a liquid chemical leak is related to the properties of the substance as well as the concentration at which the leaked substance evaporates and diffuses, and the amount of vapor generated is proportional to the surface area of the liquid [5]. Since the liquid penetrates into the surrounding soil, environmental damage caused by soil contamination and subsequent remediation requires a great deal of effort [6]. These problems can be prevented by installing a dike, which can prevent the spread of damage caused by liquid leaking from the storage facility [7]. The dike can be installed and maintained according to the regulations to prevent the spread of chemical substances, but there is a possibility that hazardous chemicals may overflow out of the dike if a large-capacity storage tank ruptures [8].

Despite international interest in improving the safety of chemical processes, several accidents have occurred. While refueling a large fuel storage tank for an oil company in Pennsylvania, USA in 1988, the storage tank suddenly collapsed completely [9]. The accident leaked nearly 3.9 million gallons of diesel fuel and more than 1 million gallons flowed into the nearby Monongahela River. As a result, about one million people living in the area were affected by the pollution, with some living without water for more than a week. A storage tank located in Michigan, USA in 1999, which held 1 million gallons of ammonium polyphosphate, ruptured, causing permanent damage to three other nearby tanks [10]. Similarly, in Ohio, USA in 2000, a tank storing one million gallons of liquid fertilizer ruptured, damaging four adjacent tanks [10]. This caused the concrete dike to rupture, pushing two excavators into the Ohio River. A total of over 800,000 gallons of liquid spilled into the river, and fertilizer mixtures were detected 100 miles downstream. In addition, two days later, a 1.5 million gallon tank of ammonium phosphate at the same facility also ruptured, destroying three nearby tanks. The discharged liquid overflowed the dike and flowed into a nearby valley. Students from nearby schools were evacuated, and there were concerns about the contamination of drinking water due to spilled chemicals [10]. Approximately 100,000 pounds (45,359 kg) of a mixture leaked from the LyondellBasell plant in Laporte, Texas, USA in 2021 from the process of manufacturing acetic acid and vinyl acetate, which is produced by combining acetic acid with ethylene and oxygen [11]. A sulfuric acid tank leak occurred in Helsingborg, Sweden in 2005, which caused problems in the bottom-to-shell weld of the steel tank, releasing 8900 m^3^ of 96% sulfuric acid over an estimated 2.5–4 min. The sudden release of the tank chemicals ruptured the roof and shell, and the acid reacted with the seawater to produce hydrogen chloride [12].

Various studies have been conducted to increase the safety of the dike installed around hazardous storage tanks to protect the surrounding buildings, machinery, instruments and facilities by preventing the chemical substances from spreading to the outside in case of leakage from the storage tank. The HSE in the UK proposed a safe and optimized methodology that was established through experiments on the ratio of the height to width of a dike in a report titled *“An experimental investigation of bund wall overtopping and dynamic pressures on the bund wall following catastrophic failure of a storage vessel”* [13]. It was intended to improve the safety of the dike by providing the research results regarding the overtopping fraction according to the capacity of the dike and the shape of the storage tank. In addition, the results obtained through modeling the tank failure in several accidents confirmed that the magnitude of the overtopping level and the dynamic pressure were high enough to cause rupture [14]. Zhang et al., (2017) studied the overtopping fractions according to the shape of the LNG and dike through mock-up tests, field tests and simulations [15].

The purpose of this study was to simulate the reduction effect of a dike installed with a deflection plate, a preventive device that can minimize the effect of overtopping in the event of a catastrophic leakage at a chemical storage facility. In addition, chemicals with different viscosities (i.e., nitric acid, sulfuric acid) were selected to compare the effect of overtopping caused by chemical leakage. Based on these research results, a safe shape of the dike that can reduce the damage caused by the discharge of chemicals due to a catastrophe was proposed, and a device to prevent additional problems was also proposed.

## 2. Materials and Methods

### 2.1. Design Selection for Simulation

Various safety regulations to prevent leakage are being implemented at chemical plants in South Korea, and damage has been minimized through these safeguards [16,17,18]. Among them, the dike prevents the chemicals in the storage facility from spreading due to a leak accident, and the deflector plate plays a role in preventing the overtopping of the leaking chemicals on the dike by instantaneous release. In this study, a simulation was performed according to storage facilities and the dike in accordance with the Hazardous Chemical Safety Management Act, which provides specific standards among various related laws [16]. The storage facility was designed to have a circular shape with a radius of 2.52 m and a height of 5.03 m with a storage capacity of 100 m^3^ and a radius and height ratio of 0.5 (radius/height). It was assumed that the storage capacity of the storage facility would be 100% filled. The capacity of the dike is the value obtained by subtracting the capacity of the tank and the structure of the dike from the capacity of the dike itself, and this was set to 110 m^3^, which is 110% of the storage facility according to the relevant standards. The thickness of the dike was also set to 0.2 m according to the legal standards.

**Figure 1:**
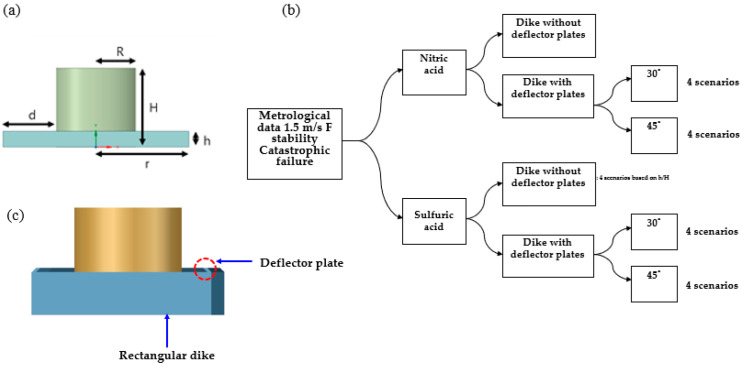
(**a**) Dimensions of the model, (**b**) design of various scenarios used in simulation, and (**c**) schematic image of a rectangular dike and deflector plate.

Greenspan and Johansson (1981) presented Equation (1) to estimate the overtopping fraction [19].

(1) $$Q=Q\;(\frac{h}{H},\;\frac{r}{H},\;\frac{R}{H},\;\theta )$$

where *Q* is the overtopping fraction, *h* is the height of the dike, *H* is the height of the storage facility, *r* is the radius of the dike, *R* is the radius of the storage facility, and θ is the slope of the dike, as shown in Figure 1a. According to Atherton’s (2005) experiment, *h*/*H* was the most important variable, followed by *r*/*H*, *R*/*H*, and θ [13]. Therefore, the dike was designed with a difference in the value of h/H. Figure 1b schematically shows various scenarios, and detailed dimensions are shown in Table 1. Six types of dike were designed by setting the ratio of the height of the dike to the height of the storage facility as 0.5, 0.4, 0.3, 0.2, 0.1, and 0.05, based on a rectangular shape. Among them, the final four scenarios were selected, except for the two scenarios that did not meet the criteria of the height of the dike and the distance from the storage facility according to the safety regulations. In addition, the safety of the dike was assessed by installing a deflection plate with a length of 0.3 m at 30° and 45° of the dike. In order to check the effect of the deflector plate according to the angle, two angles were randomly selected and compared. Figure 1c shows a schematic image of the dike and deflector plate used in the simulation. In this study, the height of the deflector plate was considered constant, and the deflector plate was designed to be located between dikes. Therefore, a total of 16 scenarios were investigated.

The chemicals used in the simulation were selected based on accident frequency. According to data from the Korea Chemical Information Service, 111 chemical accidents were identified between 2014 and 2018, and the most common chemicals were nitric acid (HNO_3_) and sulfuric acid (H_2_SO_4_) [20]. There is a large difference in the viscosity of sulfuric acid (24.2 cP) and nitric acid (1.1 cP) at 25 °C, which can affect the overtopping of the dike; therefore, the effect of viscosity was analyzed by comparing it with water.

### 2.2. Weather Conditions

The meteorological conditions can have an impact on people and the environment in the case of maximum leakage from the storage facility where the chemical is stored, and they were set as the worst-case scenario in the off-site impact assessment. A temperature of 25 °C, humidity of 50%, wind speed of 1.5 m/s, and air stability F (very stable: 0.25) grade were applied in this simulation [21,22].

### 2.3. Validation of Simulation Parameters with Other Studies

The design used for the simulation was based on studies related to the storage tank and the dike, and a validation of the simulation was performed [13,14,22]. Greenspan and Johansson (1981) performed about 50 experiments on the catastrophic failure of a storage facility located in the center of a circular dike, and their study became the basis for further studies on the correlations related to overtopping [19]. Since the model of the storage facility was only about 8 inches in diameter, this presented an issue as friction can have a significant effect when applied to the actual-size storage facility. Therefore, a large-scale experiment to reduce frictional losses was conducted by Liverpool John Moores University (LJMU). They were commissioned by the Health and Safety Executive (HSE) to simulate the catastrophic failure of a storage tank [14]. To reduce friction loss, only 1/4 of the total storage facility and dike was implemented, and the entire storage facility and dike were expressed as axisymmetric. The results of the experiment were expressed as the overtopping fractions by calculating the volume that overflowed out of the dike compared to the total amount of material remaining in the dike. The height of the storage facility, the shape of the dike, and the capacity of the dike used in the experiment were varied, and the results for the storage facility with a height of 600 mm, the circular dike, and the dike capacity of 110% were compared with the CFD simulation results. The capacity of the storage facility was selected through this verification process.

### 2.4. Geometry and Grid Settings

**Figure 2:**
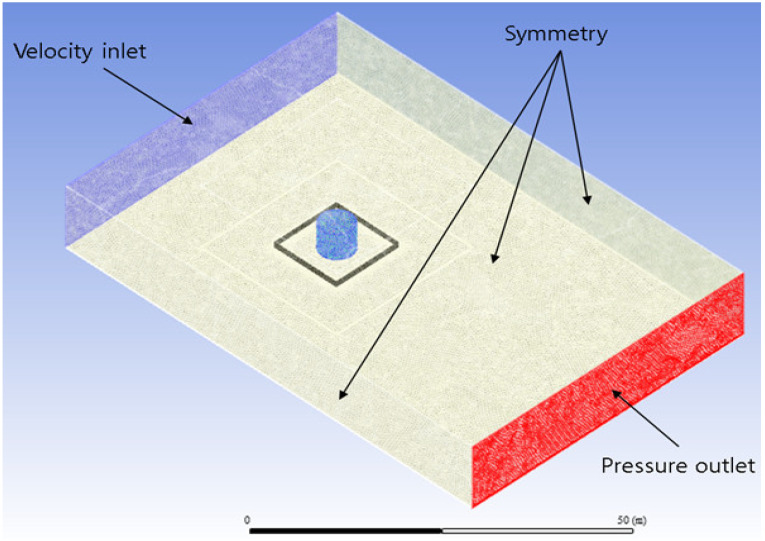
Conditions and settings of the simulation.

ANSYS SpaceClaim 19.1 and Design Modeler 19.1 (ANSYS, Inc. San Jose, CA, USA) were used to generate the geometry of the storage facility and dike. In the simulation for comparison with the experiment, the size of the entire analysis area was set to 3 m in width × 3 m in length × 1.5 m in height, and the storage facility and dike were created at the center of the width and length. The simulation was performed using the same conditions as the experiment except for the size and the contents. The dike and the external boundary were set as walls and the rest were all set as interiors. The geometric model and boundary conditions of the square bund are shown in Figure 2. The size of the entire analysis area was set to 75 m in width × 50 m in length × 12.5 m in height, and the storage facility and dike were created considering the directions of the wind blowing in and out [23]. In the simulation, it was assumed that the storage facility was installed outside the building, so the effect of wind was considered. Considering atmospheric conditions, the velocity inlet and pressure outlet were set, and both sides and the upper part were set symmetrically. In addition, the dike and the floor were set as walls, and the storage facilities were set as internal conditions.

Accuracy and analysis time are important issues in computational fluid dynamics (CFD) simulations, and both are strongly influenced by the grid [24]. After forming a tetrahedral grid using ANSYS Meshing and ANSYS Fluent, it was applied by changing it to a polyhedral grid. The tetrahedral grid is the simplest element and has the advantage of being relatively easily generated automatically compared to other grids. However, a polyhedral grid was used to obtain accurate results and to reduce the analysis time [25]. As a polyhedral mesh, the parts other than the main part were set as the growth rate to create slightly relaxed grids. The number of nodes generated in the simulation was 7,145,123, the number of faces was 8,757,336, the number of cells was 1,369,125, the minimum orthogonal quality was 0.23, and the aspect ratio was 9.

### 2.5. Numerical Analysis Model (ANSYS Fluent 19.1) and Set Conditions

An unsteady-state turbulence model and a multi-phase flow model were used in this simulation to analyze the time-dependent flow of a liquid material overtopping the dike outdoors. It is known that the turbulence equations used to simplify turbulent flow within CFD include direct numerical simulation (DNS), Reynolds averaged Navier–Stokes (RANS), and large vortex simulation (LES) [26]. A realizable *k*-*ϵ* model of quadratic equations, which is one of the RANS models, was used. The governing equation is as follows.

(2) $$\frac{\partial U_{i}\;}{\partial t}+U_{j}\frac{\partial U_{i}}{\partial x_{j}}=-\frac{1}{\rho }\frac{\partial P}{\partial x_{i}}+\nu \frac{\partial^{2}U_{i}}{\partial x_{i}\partial y_{i}}-\frac{\partial }{\partial x_{i}}\left(\bar{u_{𝚤}^{\prime }}\bar{u_{J}^{\prime }}\right)$$

$\bar{u_{𝚤}}$: mean of component velocities

$u_{i}^{\prime }$: speed of component change

ρ: density of the fluid

ν: coefficient of dynamic viscosity of the fluid

(3) $$-\left(\bar{u_{𝚤}^{\prime }}\bar{u_{J}^{\prime }}\right)=\frac{\mu_{T}}{\rho }\left(\frac{\partial u_{i}}{\partial x_{j}}+\frac{\partial u_{j}}{\partial x_{i}}\right)-\frac{2}{3}\delta_{ij}Κ$$

The Reynolds stress follows the Boussinesq hypothesis, which is to implement the modeling using the turbulent viscosity coefficient μ_T_, and is suitable for simple turbulent flow analysis such as boundary layers and mixed layers [27]. μ_T_ can be expressed as follows.

(4) $$\mu_{T}=f\left\{\frac{\rho k^{2}}{\epsilon }\right\}$$

Multiphase flow analysis was performed using a volume of fluid (VOF) model. This model was introduced by Nichols and Hirt in 1975 and is a very effective surface tracking method for multiphase flow analysis [28]. When two or more immiscible fluids exist, one equation of motion is shared among individual phases, and the volume of each phase is calculated by considering the calculated lattice volume fraction. In this method, the function F is defined as having a value of 1 at every point where the fluid exists and a value of 0 elsewhere. A cell with F = 1 is considered to be full inside, and a cell with F = 0 is considered empty. The tracing of the interface between two phases is performed with the continuity equation for the volume fraction, which is as follows [29].

(5) $$\frac{\partial F}{\partial t}+\nabla \cdot \left(uF\right)-F\left(\nabla \cdot u\right)=0$$

The VOF model has the advantage of naturally conserving volumes well, but it has a disadvantage in that it is difficult to accurately calculate the curvature or normal vector of the phase interface because it is discontinuous. Steady and transient analysis are both possible, and most calculations are made considering the gravity term. Since this method is one of the multiphase models, the phase should be defined. Usually, the phase that occupies the largest amount of the total volume is set as the primary phase and the phase that occupies the next largest volume is set as the secondary phase [30]. Table 2 summarizes the conditions used in the simulation.

## 3. Results and Discussion

### 3.1. Verification of Simulation with Experimental Data

In the actual experiment, the radius and height of the circular storage facility were 300 mm and 600 mm, respectively, and the capacity of the rectangular dike was 110% of the capacity of the storage facility [13]. At first, when t = 0, the amount of material (kg) in the dike and the amount of material overtopping the dike after the total amount (kg) were measured. Using the preceding results of the measurement, the overtopping fraction, which is the ratio of the amount of material that overflowed the dike to the initial amount of the material, was calculated. Table 3 shows the results of comparing the overtopping fractions when the dike heights were 30, 60, 120, 180, and 240 mm. It was confirmed that the ratio of the simulation result to the actual experimental result was between 0.867 and 1.229, and that the simulation had similar values to the actual experimental results. When the height of the dike was compared with 30 mm and 60 mm, the overtopping fraction differed by about 10% depending on the height of the dike. However, a difference of about 9 to 11% occurred in the subsequent section, indicating that the difference in the overtopping fraction was relatively small. Overall, the overtopping fraction decreased as the distance between the storage facility and the dike increased and the height of the dike increased. The overtopping fractions of the closest and highest dike (*r* = 497 mm, *h* = 240 mm) and the farthest and lowest dike (*r* = 1407 mm, *h* = 30 mm) were 30.73% and 69.95%, respectively, with a difference of about 2.3 times.

### 3.2. Comparison of Overtopping Fractions

**Figure 3:**
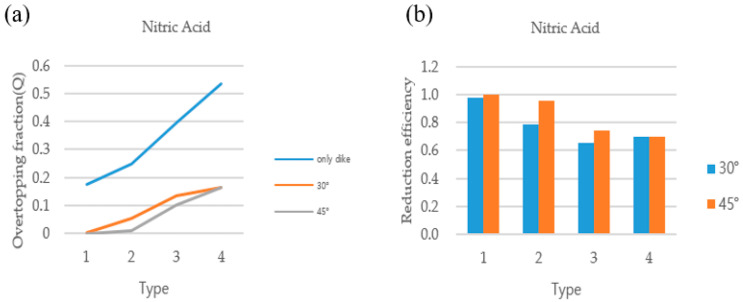
(**a**) Analysis of nitric acid overtopping fraction, and (**b**) reduction efficiency of nitric acid with deflector plates (30°, 45°) in the dike.

**Figure 4:**
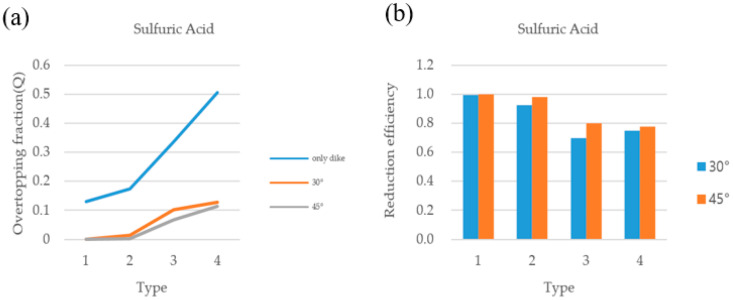
(**a**) Analysis of sulfuric acid overtopping fraction, and (**b**) reduction efficiency of HNO_3_ with deflector plates (30°, 45°) in the dike.

The overtopping fractions of the dikes that satisfy the enforcement rules of the laws and regulations related to chemical substances when all the substances in the storage facility with/without deflector plates have been released were compared. When the ratio of the radius to the height of the storage facility was 1:2, the overtopping fraction tended to decrease as the dike got closer to the storage facility and higher, regardless of the material, in both the basic dike and the dike attached with a deflector plate. The storage facility ratio applied the standards of the Dangerous Materials Safety Management Act of the Republic of Korea. Figure 3 and Figure 4 show the overtopping fractions of sulfuric acid and nitric acid when the deflector plates were attached to the dike at two different angles (i.e., 35°, 45°). In the case of a leak in the liquid tank as shown in Figure 3a, the dike with the deflector plate was very effective in reducing overtopping compared to the dike without the deflector plate, and the 45° angle deflector was more effective than the 30° deflector. Figure 3b also shows that the reduction efficiency was more than 98% in nitric acid when the deflection plate was attached to the dike at 30° and 45° angles. Sulfuric acid showed the same tendency as nitric acid, as shown in Figure 4. However, as a result of observing the overtopping fraction according to the substances (nitric acid, sulfuric acid) in the storage facility, nitric acid had a greater amount of overtopping than sulfuric acid in both dikes with/without deflector plates (30°, 45°). This is probably due to the influence of the chemical properties on the overtopping of the dike. The analysis results demonstrate that the presence of a dike with a deflection plate has a significant effect on reducing the leakage of hazardous chemicals, and Type 1 showed the best performance among the four types. It was also revealed that the dike with the 45° deflection plate showed higher efficiency than the dike with the 30° deflection plate in all cases.

**Figure 5:**
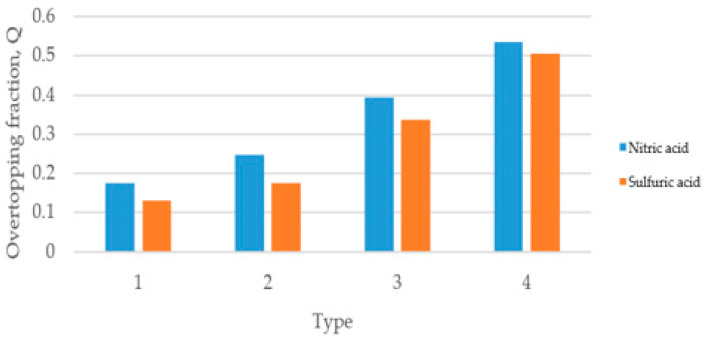
Overtopping fractions according to dike without deflector plates.

**Figure 6:**
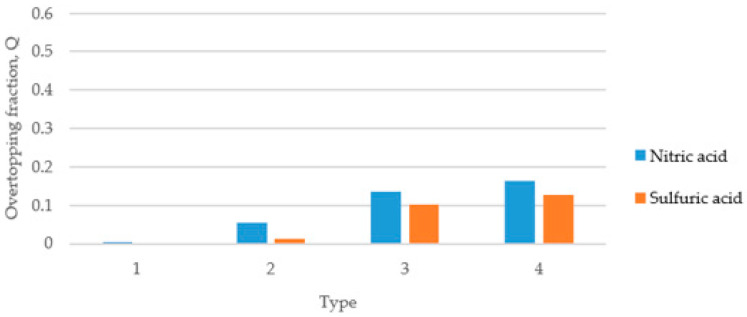
Overtopping fractions according to dike type (30°) without deflector plates.

**Figure 7:**
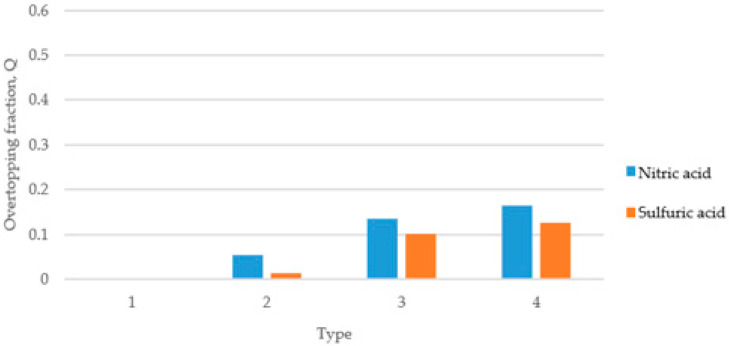
Overtopping fractions according to dike type (45°) without deflector plates.

Figure 5 shows the results of calculating the overtopping fractions when there are no deflector plates in the dike. Depending on the installation of deflector plates, a difference of up to 7% occurred in the overtopping. Additionally, nitric acid is more likely to cause overtopping of the dike than sulfuric acid, indicating that density and viscosity have a slight effect on overtopping over time. In Figure 5, Figure 6 and Figure 7, the overtopping fracture was analyzed by changing the angle of the dike without installing deflector plates. The overtopping fracture in Type 1 was the least, and the value of nitric acid was higher than that of sulfuric acid in all types. The installation of the dike at an angle of 30 degrees may help prevent overtopping, but the effect of the angle was not significant.

Three things were confirmed through the simulation. The reduction in the overtopping was different depending on whether deflector plates were installed on the dike, and it was confirmed that the efficiency of the deflector plates was better. Second, it was confirmed that the effect on overtopping was different depending on the angle of the deflector plates installed on the dike. The overtopping reduction effect of the dike with the 45° deflector plate was greater than that of the dike with the 30° deflector plate. In addition, the amount of overtopping material in the dike was greatest in Type 4, followed by Type 3, 2, and 1. Through this analysis, it was possible to confirm the appropriate dimensions of the dike when installing the deflector plates on the dike.

**Figure 8:**
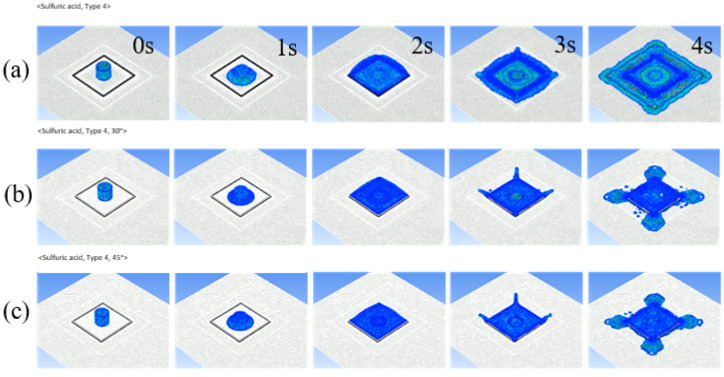
Sulfuric acid overtopping phenomena of the Type 4 dike according to time (0~4 s). (**a**) Dike without deflector plates, (**b**) dike with deflector plates with 30° angle, and (**c**) dike with deflector plates with 45° angle.

**Figure 9:**
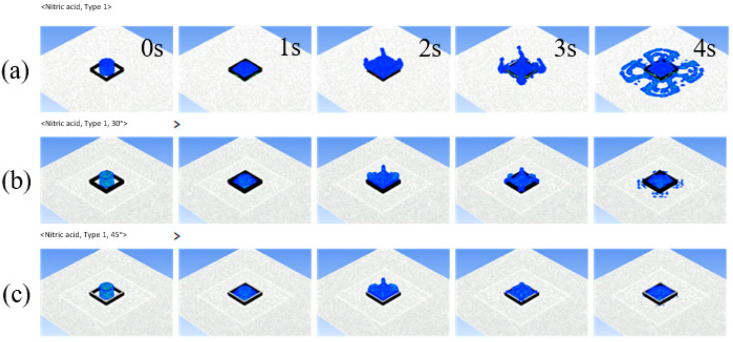
Nitric acid overtopping phenomena of the type 1 dike according to time (0~4 s). (**a**) Dike without deflector plates, (**b**) dike with deflector plates with 30° angle, and (**c**) dike with deflector plates with 45° angle.

Figure 8 shows the phenomenon of sulfuric acid overtopping according to time (from 0 to 4 s) that occurred in the Type 4 dike, which was found to generate the most overtopping. Overtopping occurred over time, but it was shown that it started after 3 s in most cases. In the simulation, leakage due to holes was not considered because the catastrophic rupture of the tank facility was considered from the beginning of the accident. The setting of this simulation took into consideration the fact that there are more chemical accidents due to catastrophic rupture than leaks due to holes or tears in the tank facility. Figure 9 shows the overflowing phenomenon of nitric acid in the T1 type, which provides the best conditions to reduce overtopping given the dimensions of the dike. Nitric acid has a higher accident frequency in South Korea and is more likely to cause overtopping compared to sulfuric acid, and it was shown that overtopping was reduced when the Type 1 dike with deflector plates attached at an angle of 45 degrees was used. In Figure 9c, the reduction effect of overtopping is clearly shown, and it was confirmed that overtopping hardly occurred at 4 s.

## 4. Conclusions

A dike that satisfies the safety regulations and a dike with a deflection plate were designed by using computational fluid dynamics (CFD). It was confirmed that the angle affects overtopping when deflector plates are installed on the dike, and installing deflector plates on the dike helps to reduce overtopping. In addition, the chemical substances that are most frequently involved in accidents were selected, and it was confirmed that the density and viscosity of the chemical substances had an effect on overtopping. However, there are limitations in not verifying the effect of the height of the deflector plate and the usefulness according to the scale-up in the simulation. The detailed results obtained through simulations are as follows.

The dike showed a tendency to decrease the overtopping fraction when the dike was closer to the storage facility and the height of the dike increased, regardless of the leaking chemicals.
The simulation showed that when the ratio of the radius to the height of the storage facility was 1:2, the dike with the deflection plate (30°, 45°) showed an excellent reduction efficiency of 98~99%.
When the storage capacity of the dike was 110% of the maximum capacity of the storage facility, the dike with the deflection plate (30°, 45°) was more effective in reducing the overtopping phenomenon than the dike without the deflection plate.
Compared with the dike without deflector plates, the reduction efficiency of the dike with 30° and 45° deflector plates ranged from 65 to 99% and 69 to 99%, respectively.
The dike with a 45° deflection plate showed greater reduction efficiency, which ranged from a minimum of 0.35% to a maximum of 17.45%, than that of the dike with a 30° deflection plate.
In all types of dikes in this study, nitric acid exhibited overtopping phenomena that was up to about 7% higher than sulfuric acid, indicating that the density and viscosity of the material affected the overtopping fractions over time, although the differences were not large.
It is necessary to install additional safety devices at the junction between the dike and the deflection plate to withstand the pushing pressure that builds up when the liquid overflows the dike.

It should be noted that the overtopping reduction efficiency may vary if there is a change in the shape, such as increasing or lowering the height of the storage facility. Through simulation, it was confirmed that the overtopping reduction efficiency is higher if the deflection plate is attached to the dike and the deflection plate is attached to the dike at an angle of 45°. Therefore, in worst-case scenarios such as disasters, attaching a 45° deflection plate to the dike is an effective way to prevent overtopping from chemicals that spread to the outside after the dike overflows. When the deflector plate is attached, it is necessary to install an additional fixing device at the junction between the dike and the deflector plate to withstand the pressure exerted by the liquid overflowing the dike.

## Figures and Tables

**Table 1:** Dimensions of the storage facility and dike.

Dimension	Value			
Tank volume (V_t_ (m^3^))	100			
Dike capacity (V_d_ (m^3^))	110			
Dike thickness (m)	0.2			
*R* (m)	2.52			
*H* (m)	5.03			
Type	1	2	3	4
*h*/*H*	0.4	0.3	0.2	0.1
*h* (m)	2.012	1.509	1.006	0.503
*r* (m)	4.319	4.818	5.685	7.724
d (m)	1.799	2.298	3.165	5.204

h: height of the dike; H: height of the storage facility; r: the radius of the dike; R: radius of the storage facility; d: distance between the dike wall and the surface of the storage tank.

**Table 2:** CFD analysis conditions.

Set Conditions	Validity Verification	Case Studies	
Turbulence model	Realizable *k*-*ϵ*		
Multiphase flow model	Volume of Fluid (VOF)		
Analysis method	Semi-Implicit Method for Pressure-Linked Equation (SIMPLE)		
Spatial difference	Second order upwind		
Target substance	Water	Nitric acid	Sulfuric acid
Analysis time		4 s	
Time step size	0.01 s		0.01 s
Number of time step	400		400

**Table 3:** Comparison of experimental and simulation results.

**R = 300 mm**					**Ratio** **(Simulation/** **Experimental)**
***H*** **(mm)**	***r*** **(mm)**	***h*** **(mm)**	* *Q* (%)	***Q*** **(%)**	
			**Experimental**	**Simulation**	
600	1407	30	70	69.947	0.999
600	995	60	70	60.684	0.867
600	704	120	49	49.481	1.01
600	574	180	34	39.416	1.159
600	497	240	25	30.734	1.229

* $Q=Q\left(\frac{h}{H},\frac{r}{H},\frac{R}{H},\theta \right)$ [19]; Q: overtopping fraction; h: height of the dike; H: height of the storage facility; r: the radius of the dike; R: radius of the storage facility; θ: slope of the dike.

## Data Availability

Not applicable.
